# Arsenic in Cutaneous Diseases

**Published:** 1849-11

**Authors:** 


*Arsenic in Cutaneous Diseases.*—Arsenic should never be given when there is any inflammatory action about the system, otherwise this state is sure to be increased; all the functions must first be brought into a healthy condition.

Whatever be its modus operandi in the cure of skin disease, my experience goes decidedly to establish the tonic action of arsenic. Under its use alone and without any alteration in diet or residence, I have seen marked improvement, not only in the disease, but in the general health of the patient; these, in fact, seem to go hand in hand. Herein is shown its specific action, as distinguished from other tonics; these, when exhibited, cause improvement of the health, but exert no apparent influence on the eruption. I am aware that in this opinion I differ from one of our chief authorities, Dr. Pereira, who, though he has tried it extensively, and with his attention directed to this point, has been unable to satisfy himself of its right to be classed as a tonic; the appetite of his patients sometimes improved while taking it, in no other respect was its tonic action shown. Vogl, (Pharmakodyn,) and many others, have arrived at the opposite result.

And here I would advert to the common mode of giving arsenic—i. e. in increasing doses. What tonic could be given in such a manner without danger of producing severe constitutional disorder? It is the peculiarity of this class of medicines, that their action is so gradul as to be almost imperceptible, days, or perhaps weeks, being required to show any decided effect. Thus is it with arsenic; though its action is more manifest from the complaint being external. Arsenic ought, therefore, to be given in moderate, or what might be called tonic doses, at first until the system comes under its influence; these must be diminished, if it affects the patient too severely. The action of arsenic seems to be more direct on the tegumentary tissues, the skin, and mucous membranes, and its influence is nearly always first manifested on the tarsal conjunctiva; hence we have a ready guide to its exhibition. The degree at which we wish to arrive, is a very slight injection of the membrane; if it goes beyond that point, and more severe irritation is caused, then the eruption becomes inflamed, and even spreads; this may in almost every case be prevented by diminishing the dose.—If the system be kept in this state, no injurious operation need be feared. Five minim doses of Fowler's solution may be safely given at first to a person who is moderately strong, has not had its constitution injured by its abuse and who is not unusually intolerant of it. Cases will, however, present themselves to the practitioner, in which it will be advisable to begin with a smaller dose than this; indeed, if there be any doubt, it is better to give the smaller dose, two, three, or four minims, and pursue it for a longer time; the system is sure, though at a later period, to come under its power. The disease is generally seen to date its improvement from the time of the first affection of the conjunctiva.

Again, arsenic should be taken with the meals, or on a full stomach; this serves to dilute it, and prevents the gastric irritation which so frequently follows when it is exhibited without this precaution.

The decided influence which this remedy has over certain eruptive disorders, is shown not merely by their amelioration under its proper use, but more manifestly by omitting it for a time, as I have frequently done, and invariably has the disease recommenced spreading as soon as the system had got rid of the arsenic, as shown by the subsidence of the conjuctivitis, and its course has been again stayed after the medicine has been resumed.

